# Histone-fold centromere protein W (CENP-W) is associated with the biological behavior of hepatocellular carcinoma cells

**DOI:** 10.1080/21655979.2020.1787776

**Published:** 2020-07-07

**Authors:** Ziliang Zhou, Zhechong Zhou, Zhaoxia Huang, Suhua He, Shoudeng Chen

**Affiliations:** a Molecular Imaging Center, Guangdong Provincial Key Laboratory of Biomedical Imaging, The Fifth Affiliated Hospital, Sun Yat-sen University, Zhuhai, China; b Department of Experimental Medicine, The Fifth Affiliated Hospital, Sun Yat-sen University, Zhuhai, China

**Keywords:** Centromere protein W, hepatocellular carcinoma, predictive biomarker, RNA-seq, bioinformatics

## Abstract

Centromere protein W (CENP-W), identified as a centromeric component, plays an important role in the cell life cycle. However, how *CENPW* expression affects biological processes in liver cancer cells remains unknown. In this article, we found that *CENPW* was overexpressed in liver cancer tissues. Low *CENPW* expression was correlated with a better prognosis in hepatocellular carcinoma (HCC) patients, compared to high *CENPW* expression. The results of qRT-PCR and western blot assay showed that *CENPW* was effectively knocked down in HCC cells using siRNA transfection. Cell proliferation, migration, and invasion were inhibited. Cell apoptosis rates were increased. The cells were arrested in the G2/M phase of the cell cycle. Subsequently, 127 differentially expressed genes (DEGs) were identified based on RNA-seq data. GO and KEGG enrichment and PPI network analysis were performed. The novel DEGs were found and mainly enriched in nucleosome assembly and the complement system. In summary, our study indicated that overexpression of *CENPW* implied unfavorable prognosis and *CENPW* might be the potential predictive biomarker in liver cancer. Downregulation of *CENPW* might inhibit the HCC developmentby regulating the expression of the molecules in nucleosomes and the complement system.

## Graphical abstract

**Figure:**
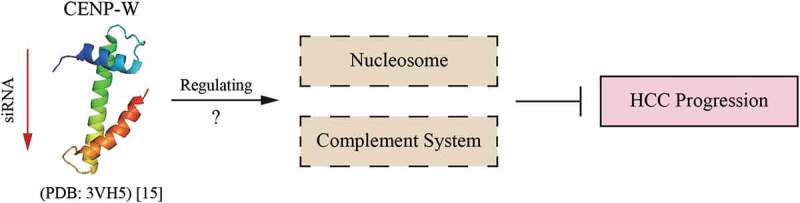

## 1. Introduction

HCC is the most common primary liver cancer. Worldwide, liver cancers are the fourth leading cause of cancer mortality and rank sixth in the incidence of various cancers [1]. Approximately 80% of liver cancer cases are caused by chronic infections with hepatitis b virus (HBV) and hepatitis c virus (HCV) [2]. In Western countries and Japan, HCV infection is the main cause of HCC. However, in Asia and sub-Saharan Africa, HBV infection becomes the main cause of HCC [1,3]. The five-year survival rate of liver cancer is only 18% and is as low as 12% in Asian countries such as China. The high recurrence and metastasis of HCC are the main causes limiting long-term survival [4,5]. Liver resection is currently the most effective way to treat HCC at an early stage [6,7]. When the patient is at the intermediate stage or advanced stage of HCC, systemic therapies are usually difficult to avoid. However, liver cancer has high molecular heterogeneity, stimulating tumor evolution, which can drive resistance to systemic therapies [8,9]. In the clinic, α-fetoprotein (AFP) is most widely used as a biomarker for HCC, but only to bean auxiliary diagnostic index because of its low specificity and sensitivity [10]. According to Agopian’s study, out of 665 HCC patients, 31.3% had non-AFP-producing tumors [11]. It is extremely urgent to improve therapeutic strategies and discover new diagnostic biomarkers for HCC.

Centromere protein W (CENP-W), which used to be called cancer-upregulated gene 2 (CUG2) protein, is overexpressed in various human cancers. Encoded by the *CENPW* gene, CENP-W is identified as a centromeric component [12,13]. CENP-W can form a DNA-binding heterodimer together with CENP-T [14]. Further, CENP-T-W and CENP-S-X complexes are packed into a stable CENP-T-W-S-X heterotetramer with a histone-like fold, forming a unique centromeric chromatin structure that allows them to interact with DNA. The CENP-T-W-S-X complex was identified to bind to approximately 100 bp DNA by an MNaseprotection assay and is a nucleosome-like structure [15]. Later, biochemical experiments revealed that the CENP-T-W-S-X complex preferentially binds to dinucleosomes with 100 bp linker DNA as a (CENP–T–W–S–X)_2_ structure [16]. Such 100 bp binding regions for the CENP-T-W-S-X complexes might be interspersed at centromeres between canonical nucleosomes [17]. However, we found that only CENP-W was overexpressed in liver cancer through bioinformatics analysis, while CENP-T-S-X were not. Moreover, it was reported that CENP-W can interact with proteins such as nucleophosmin (NPM) [12] and enhancer of zeste homolog 2 (EZH2) [18] and facilitate chromatin transcription (FACT) in the nucleus [19]. We were curious about the cellular function of CENP-W in the course of our study about HBV covalently closed circular DNA (cccDNA) nucleosomes. We hypothesized that CENP-W might have biological function beyond its role as a component in a nucleosome-like structure.

*CENPW* was reported to be upregulated in many human cancer tissues, and exogenous *CENPW* overexpression was shown to induce tumorigenesis. Recent cellular experiments have mostly focused on the overexpression of exogenous *CENPW* [20]. In contrast, knocking down endogenous *CENPW* has scarcely been reported. In addition, there are no data about the function of *CENPW* in HCC cells. Therefore, we explored the downregulation of the *CENPW* gene, seeking the potential diagnostic biomarker and therapeutic target for HCC. In our study, we firstly confirmed the overexpression of the *CENPW* gene in various cancer tissues including HCC via analysis of the Oncomine database. Survival analysis was then carried out by using the Kaplan-Meier plotter database, showing the correlation between *CENPW* expression and the prognosis of liver cancer. Next, endogenous *CENPW* expression of human HCC cells was knocked down by siRNA transfection. Real-time quantitative reverse transcriptase-polymerase chain reaction (qRT-PCR) and western blot were carried out to verify the downregulation of *CENPW* expression. Cell cycle, apoptosis, viability, migration, and invasion assays were performed, indicating the relationship between *CENPW* expression and tumor cell behavior. Further, RNA-seq was used to discover the downstream regulation of *CENPW* to explore the potential molecular mechanism. Overall, our study provided a clue for the biological behavior of HCC cells after knocking down *CENPW* expression, implying *CENPW* as a potential diagnostic biomarker and the target for HCC gene therapy.

## 2. Materials and methods

### 2.1. Oncomine database analysis

The mRNA differential expression level of the *CENPW* gene in different cancers was analyzed in the Oncomine 4.5 database (https://www.oncomine.org). The threshold was set as follows: *P*-value of 0.0001, fold change of 2, and gene rank of top 10%. Further, *CENPW* expression in HCC tissues was compared to that in normal liver tissues, and the differential expression with *P* < 0.01 was considered statistically significant. The analysis was based on the HCC studies of Chen Liver [21] and Wurmbach Liver [22]. Moreover, *CENPW* expression was evaluatedin subgroups of HCC patients, based on gender, age, cancer stages, and tumor grade, using UALCAN (http://ualcan.path.uab.edu).

### 2.2. Kaplan-Meier plotter database analysis

The correlation between survival and *CENPW* expression in liver cancer was analyzed by using the Kaplan-Meier plotter database (http://www.kmplot.com/analysis/). According to the median values of RNA sequencing data, liver cancer patients were split into the low and high *CENPW* expression groups. The overall survival (OS) and relapse-free survival (RFS) analyses were performed on 364 and 313 patients, respectively. Kaplan-Meier plots were obtained. Differences with *P* < 0.01 were considered significant. Log-rank *P*-values and hazard ratios (HRs) with 95% confidence intervals (CIs) were calculated.

### 2.3. Cell culture

The human hepatocellular cell line Hep3B was purchased from American Type Culture Collection (ATCC, Manassas, VA, USA), and Huh7 was kindly provided by Prof. Hong Shan (The Fifth Affiliated Hospital, Sun Yat-sen University, China). Hep3B and Huh7 cells were maintained in high-glucose Dulbecco’s modified Eagle’s medium (DMEM, Gibco, Grand Island, NY, USA) supplemented with 10% fetal bovine serum (FBS, MoregateBiotech, Bulimba, Queensland, Australia) and 1% penicillin streptomycin (Gibco) in a humidified incubator (Thermo Fisher Scientific, Waltham, MA, USA) with 5% CO_2_ at 37°C.

### 2.4. Cell transfection

Four pairs of siRNAs (siRNA-539, siRNA-712, siRNA-758, and siRNA-900) targeting *CENPW* and negative control siRNA-NC were synthesized (GenePharma Co. Ltd., Shanghai, China) and purified by high-performance liquid chromatography (HPLC). The siRNAs were transfected by using Lipofectamine^TM^ 3000 Transfection Reagent (Invitrogen, Carlsbad, CA, USA). After transfection for 24 to 48 h, cells were used for subsequent experiments.

### 2.5. RNA preparation and qRT-PCR

Using the FastPure Cell/Tissue Total RNA Isolation Kit (Vazyme Biotech, Nanjing, China), total RNA was extracted from Hep3B and Huh7 cells. The purity and concentration of the total RNA were detected using a Nanodrop 2000 Spectrophotometer (Thermo Fisher Scientific). HiScriptIII RT SuperMix for qPCR (+gDNA wiper) (Vazyme Biotech) was applied for reverse transcription to synthesize cDNA. qPCR was performed using ChamQ Universal SYBR qPCR Master Mix (Vazyme Biotech) and CFX96 Touch Real-Time PCR Detection System (Bio-Rad, Hercules, CA, USA). The β-actin gene was selected as the internal control against the *CENPW* gene. The primers were as follows: for the β-actin gene:5ʹ-AGGTGGACAGCGAGGCCAGGAT-3ʹ (forward), 5ʹ-TTGCCGACAGGATGCAGAAGGA-3ʹ (reverse); and for the *CENPW* gene: 5ʹ-TAGCAGAAGAGTCCAGGACAAAC-3ʹ (forward), 5ʹ-ACCTCTGCTCTTCTTTAGAATTACC-3ʹ (reverse). *CENPW* expression was normalized to β-actin expression, and relative expression was calculated using the 2^−ΔΔCt^ method.

### 2.6. Western blot analysis

HCC cells were harvested after 48 h of transfection and lysed by RIPA Lysis Buffer (Beyotime, Shanghai, China). After 14,000 × g for 10 min at 4°C, total protein concentration was measured by the Enhanced BCA Protein Assay Kit (Beyotime). The protein was separated by 18% SDS-PAGE and transferred to a PVDF membrane. The membrane was blocked by 5% skim milk and incubated with the primary antibodies against CENP-W (ab75827, Abcam, Cambridge, MA, USA) and GAPDH (ab181602, Abcam). After incubation with the secondary antibody, the bands were detected with enhanced chemiluminescence. The density of the blots was measured by ImageJ version 1.51. CENP-W expression was normalized to GAPDH expression, and relative protein expression was calculated.

### 2.7. Cell viability assay

After transfection for 48 h, Hep3B cells and Huh7 cells were inoculated into 96-well plates with 100 μl complete medium (5,000 cells/well). Then, 10 μl of CCK-8 (APExBIO, Houston, TX, USA) solution was added into each well at 0, 24, 48 and 72 h. After 2 h incubation, optical density (OD) was measured at a 450 nm wavelength by a microplate reader (BioTek, Winooski, VT, USA). Cell proliferation curves were calculated and plotted.

### 2.8. Transwell assay

Transwell migration and invasion assays were performed using 24-well chambers with 8 μm pore-size membranes (Corning, NY, USA). After transfection for 48 h, serum-free medium was used to starve the cells for another 12 h. Then, 6 × 10^4^ cells were inoculated into the upper chamber in 200 μl of serum-free medium, and 600 μl of complete medium with 10% FBS was placed into the lower chamber. Transwell chambers were coated with Matrigel (Corning) only for the invasion assay. After 24 h incubation at 37°C, cells were fixed with 4% paraformaldehyde for 30 min at room temperature and stained with 0.1% crystal violet overnight. The nonmigrating cells were removed before the cells on the lower surfaces of the inserts were counted under a microscope (Leica, Wetzlar, Germany).

### 2.9. Cell apoptosis assay

After 48 h of transfection, Hep3B and Huh7 cells were harvested and washed with cold phosphate buffered saline (PBS, Gibco) twice. Using the Annexin V-FITC/PI Apoptosis Detection Kit (Vazyme Biotech), 5 × 10^5^ cells were resuspended in 100 μl 1 × binding buffer and then stained with 5 μl annexin V-fluorescein isothiocyanate (FITC) and 5 μl propidium iodide (PI) for 10 min in the dark at room temperature. Another 400 μl 1 × binding buffer was added after incubation. Cells were detected on a flow cytometer (Beckman Coulter, Brea, CA, USA) within 1 h. Data were managed in FlowJo version 10.4.

### 2.10. Cell cycle assay

After siRNA transfection for 48 h, 5 × 10^5^ cells were harvested for cell cycle analysis. Cells were washed with cold PBS twice and fixed with 70% ethanol at −20°C overnight. Then, the cells were washed in PBS to remove ethanol and stained with 500 μl PI/RNase staining buffer (BD Biosciences, Franklin Lakes, NJ, USA). After incubation for 15 min in the dark at room temperature, the cells were detected on a Beckman Coulter flow cytometer. Cells were analyzed on a flow cytometer within 1 h. Data were analyzed using FlowJo version 10.4.

### 2.11. RNA-seq analysis

Total RNA was extracted from the siRNA-NC group and the siRNA-758 group of Huh7 cells after siRNA transfection for 48 h and sent to the Beijing Genomics Institute (BGI, China). Following quality control, total RNA was performed to mRNA library construction and subjected to the BGIseq500 platform (BGI) for RNA-seq detection. The raw sequencing data were filtered and the clean reads were mapped to the human genome. After that, a database was built and analyzed by Dr. Tom online system (https://report.bgi.com,BGI). DEGs were identified with |Log2 fold-change (FC)| ≥0.6 and False Discovery Rate (FDR) ≤ 0.05. Gene Ontology (GO, http://geneontology.org)biological process and Kyoto Encyclopedia of Genes and Genomes (KEGG, https://www.kegg.jp) pathway enrichment analysis were performed. The PPI network was established using theSearch Tool for the Retrieval of Interacting Genes/Proteins (STRING, https://string-db.org) and Cytoscape version 3.7.2. As previously described, the DEGs were randomly selected for qRT-PCR to validate the RNA-seq data.

### 2.12. Statistical analysis

The results of *CENPW* expression generated in the Oncomine database were displayed with gene ranks, fold changes and *P*-values. Survival curves generated by the Kaplan-Meier plotter database were displayed with HRs and *P*-values from the log-rank test. The experimental data were shown as the mean ± standard deviation (SD) from at least three replicates and were analyzed by one-way analysis of variance (ANOVA) or independent sample t-test with SPSS version 20.0. A value of *P* < 0.05 was considered statistically significant.

## 3. Results

### 3.1. *Differential expression of* CENPW

**Figure 1.**
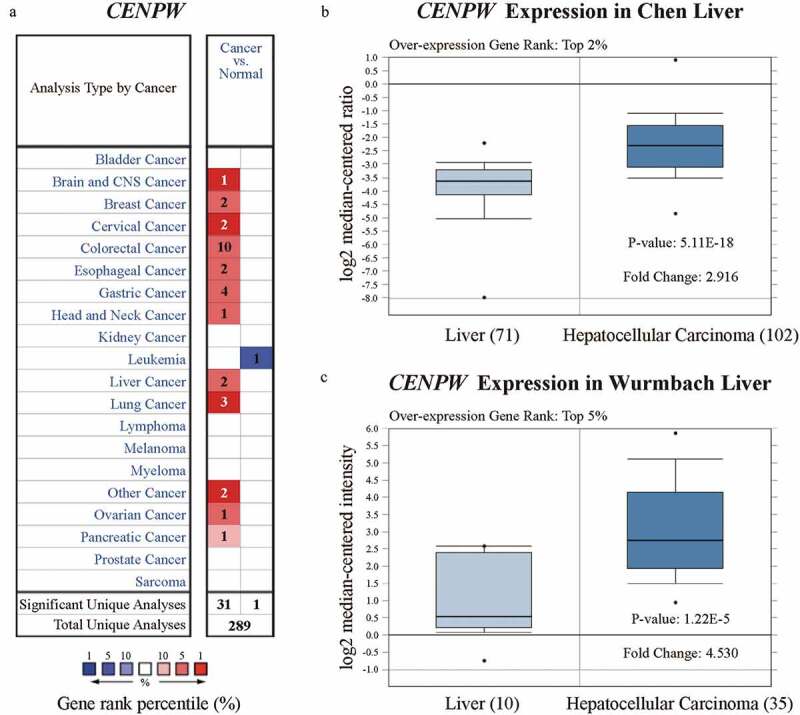
Analysis of *CENPW* differential expression in the Oncomine database. (a) *CENPW* expression levels in different types of human cancers compared to normal tissues. Upregulation is shown in red, while downregulation is shown in blue. (b, c) Box plot showing *CENPW* mRNA levels in the Chen Liver and Wurmbach Liver datasets.

We assessed the differential expression of *CENPW* by using the Oncomine database. Compared to those in normal tissues, *CENPW* expression levels were higher in various types of cancers, including brain and central nervous system (CNS) cancer, breast cancer, cervical cancer, colorectal cancer, esophageal cancer, gastric cancer, head and neck cancer, liver cancer, lung cancer, ovarian cancer, pancreatic cancer and other cancers (Figure 1(a)). Considering all the cancers above, *CENPW* was downregulated only in leukemia. We further analyzed *CENPW* mRNA expression in HCC tissues and normal liver tissues and found that the expression level was significantly higher in HCC tissues (*P* < 0.01). The fold change was within 2.916, and the overexpression gene rank was within the top 2% in Chen Liver (Figure 1(b)), while the fold change was within 4.530, and the rank was within the top 5% in Wurmbach Liver datasets (Figure 1(c)). We also analyzed *CENPT, CENPS* and *CENPX* expression, but no significant difference was found between liver cancer tissues and normal tissues (data not shown), implying that CENP-W might play roles other than its role as a component in a nucleosome-like structure. Further, using UALCAN in subgroup analyses, we confirmed that the transcription level of *CENPW* was significantly higher in HCC patients than that in normal people, including the early stage of HCC (Figure S1). Notably, in the subgroup of cancer stages, when compared to the stage 1, the *CENPW* expression of stage 2 (*P* = 0.0064 < 0.01) and stage 3 (*P* = 0.0002 < 0.001) were also significantly increased (Figure S1C). These results implied the expression of *CENPW* might isolate well the early stage of HCC and be a potential biomarker in HCC early diagnosis.

### 3.2. Prognostic potential of CENPW in liver cancer

**Figure 2.**
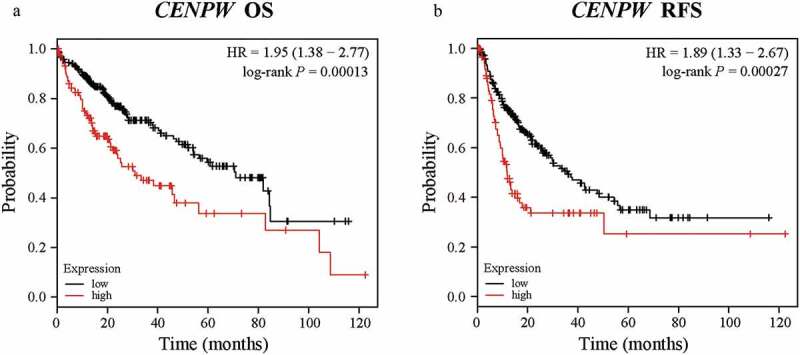
Kaplan-Meier survival analysis comparing the low and high expression of *CENPW* in patients with liver cancer in the Kaplan-Meier plotter database. (a) Survival analysis of overall survival (OS) in the liver cancer cohort (*P* < 0.01). (b) Survival analysis of relapse-free survival (RFS) in the liver cancer cohort (*P* < 0.01).

The Kaplan-Meier plotter database can perform the survival analysis of approximately 54,000 genes in 21 cancer types. The data are derived from the Gene Expression Omnibus (GEO), European Genome-phenome Archive (EGA) and The Cancer Genome Atlas (TCGA) databases. To investigate whether *CENPW* expression was correlated with prognosis in liver cancer patients, we used the Kaplan-Meier plotter database to evaluate the prognostic potential of *CENPW* based on the RNA sequencing data. Notably, poor prognosis in liver cancer (OS HR = 1.95, 95% CI = 1.38 to 2.77, log-rank *P* = 0.00013; RFS HR = 1.89, 95% CI = 1.33 to 2.67, log-rank *P* = 0.00027) was correlated with high *CENPW* expression (Figure 2). The results indicated the ability of *CENPW* to predict unfavorable prognosis in liver cancer, showing its value as a diagnostic biomarker.

### 3.3. CENPW mRNA expression in HCC cells

**Figure 3.**
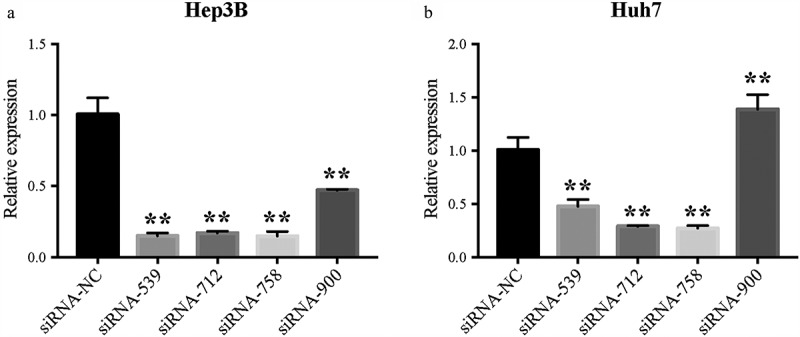
*CENPW* mRNA expression was knocked down after siRNA-539, siRNA-712, or siRNA-758 transfection in (a) Hep3B and (b) Huh7 cells. The relative expression of *CENPW* was significantly downregulated (**P < 0.01) compared to the siRNA-NC group.

The relative *CENPW* mRNA expression in Hep3B and Huh7 cells was calculated after transfection for 24 h with the siRNAs (Figure 3). *CENPW* mRNA expressions in the siRNA-539, siRNA-712 and siRNA-758 group were significantly decreased compared to that in the siRNA-NC group (*P* < 0.01). On the whole, siRNA-758 exhibited the best knockdown efficiency in both HCC cells and its data showed 14.81% ± 3.26% free *CENPW* mRNA in Hep3B cells and 27.14% ± 2.41% in Huh7 cells by normalizing to the siRNA-NC group as a negative control. Therefore, we chose siRNA-758 for subsequent experiments.

### 3.4. CENP-W protein expression in HCC cells

**Figure 4.**
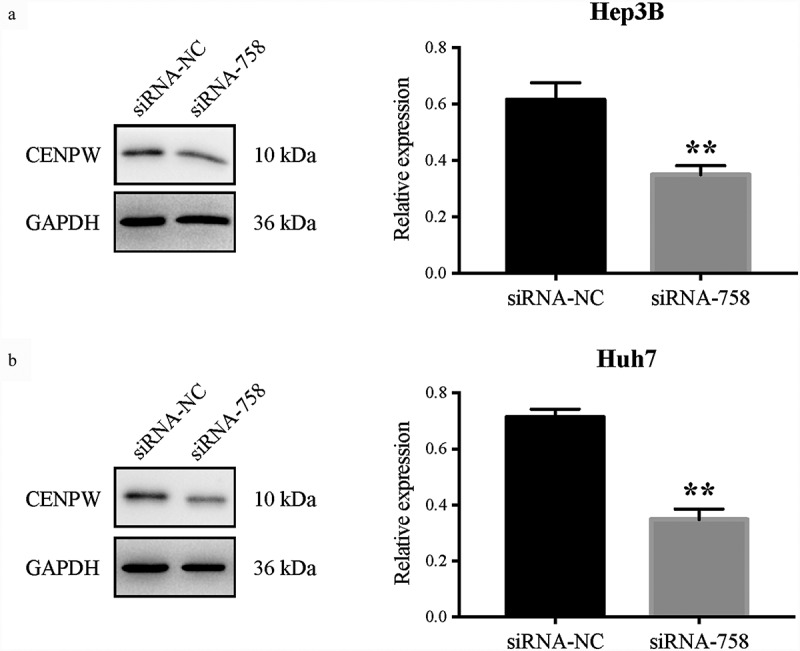
CENP-W protein expression level was significantly downregulated in (a) Hep3B and (b) Huh7 cells (**P < 0.01) after siRNA-758 transfection when compared to the siRNA-NC group.

The CENP-W expression in protein level was detected by western blot assay (Figure 4). Compared to the siRNA-NC group, the CENP-W expression was significantly downregulated in both Hep3B and Huh7 cells (*P* < 0.01). The results confirmed that the siRNA-758 transfection knocked down the CENP-W protein expression level.

### 3.5. CENPW influences proliferation in HCC cells

**Figure 5.**
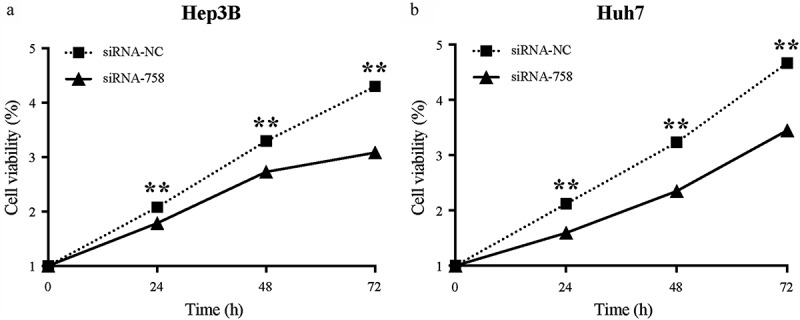
Knocking down *CENPW* influenced cell proliferation in (a) Hep3B and (b) Huh7 cells. The CCK-8 assay indicated that the cells in the siRNA-758 group grew significantly slower than those in the siRNA-NC group (**P < 0.01).

The proliferation of Hep3B and Huh7 cells was examined continuously for 72 h by the CCK-8 assay. The results showed that the proliferation in both cell lines was obviously slowed (*P* < 0.01) from 24 to 72 h in the siRNA-758 group compared to the siRNA-NC group (Figure 5). Thus, knocking down *CENPW* decreased the proliferation ability of HCC cells, indicating that *CENPW* overexpression might be the cause of uncontrolled proliferation.

### 3.6. CENPW affects the migration and invasion of HCC cells

**Figure 6.**
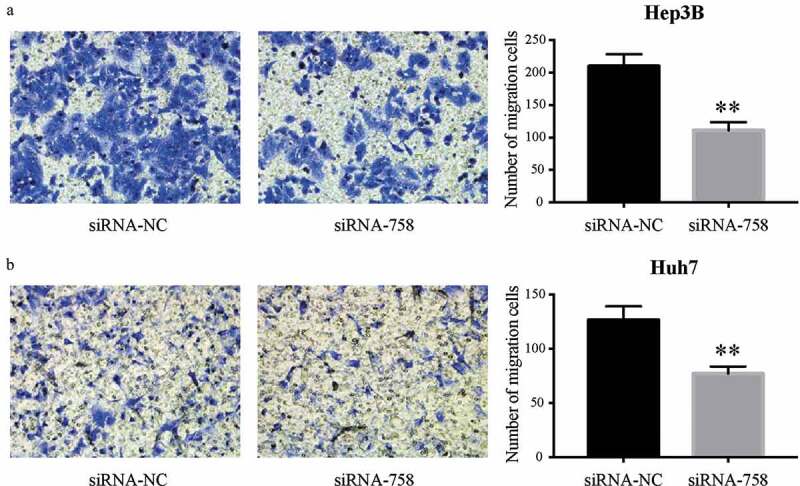
Knocking down *CENPW* inhibited cell migration in (a) Hep3B and (b) Huh7 cells. The Transwell assay showed that the number of migrated cells in the siRNA-758 group was obviously reduced compared to the siRNA-NC group (**P < 0.01).

**Figure 7.**
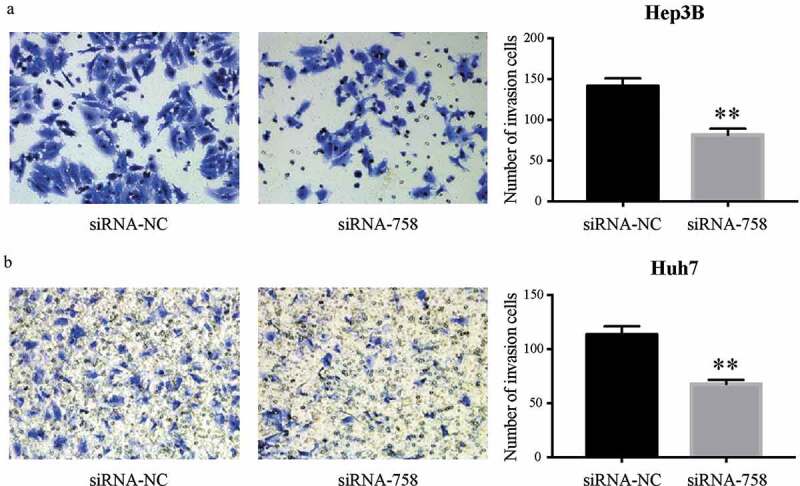
Knocking down *CENPW* expression suppressed cell invasion in (a) Hep3B and (b) Huh7 cells. The Transwell assay indicated that the invasion of HCC cells in the siRNA-758 group was significantly inhibited compared to that in the siRNA-NC group (**P < 0.01).

The motility and aggressiveness of Hep3B and Huh7 cells were examined by the Transwell assay. The migrating cells were significantly reduced (*P* < 0.01) in the siRNA-758 group, indicating the contribution of *CENPW* to cell motility (Figure 6). Similarly, the number of invading cells was obviously decreased (*P* < 0.01) in the siRNA-758 group, showing that *CENPW* confers aggressive features in HCC cells (Figure 7). Knocking down *CENPW* reduced the migration and invasion abilities of HCC cells.

### 3.7. CENPW influences cell apoptosis

**Figure 8.**
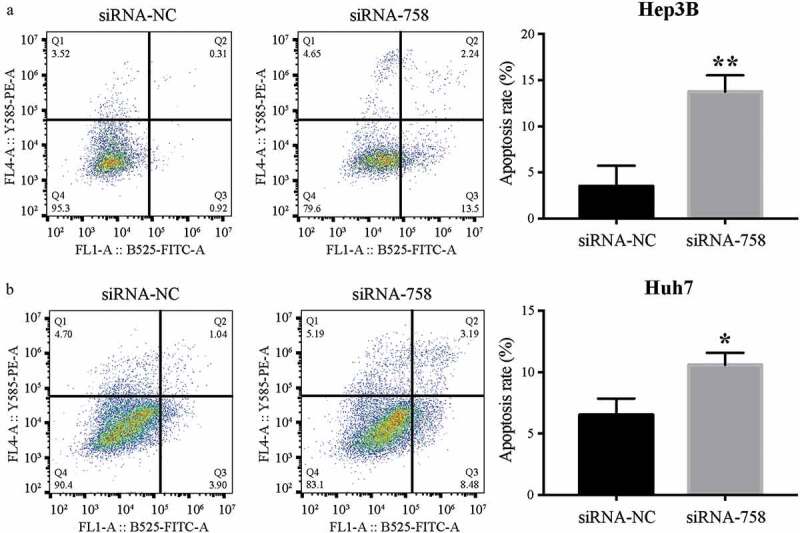
The distribution of apoptosis in (a) Hep3B and (b) Huh7 cells was examined by flow cytometry. The apoptosis rates in the siRNA-758 groups were significantly increased in Hep3B (**P < 0.01) and Huh7 (*P < 0.05) cells.

The apoptosis assay showed that the apoptosis rates of Hep3B and Huh7 cells were 3.48% ± 2.25% and 6.50% ± 1.35% in the siRNA-NC group, while the rates were 13.74% ± 1.79% and 10.59% ± 0.98% in the siRNA-758 group. The apoptosis rates were significantly increased by siRNA-758 transfection in Hep3B (*P* < 0.01) and Huh7 (*P* < 0.05) cells (Figure 8). The results indicated that knocking down *CENPW* expression could induce HCC cell apoptosis. In addition, the expression of *CENPW* might be important in the development of HCC cells.

### 3.8. CENPW influences the distribution of the cell cycle

**Figure 9.**
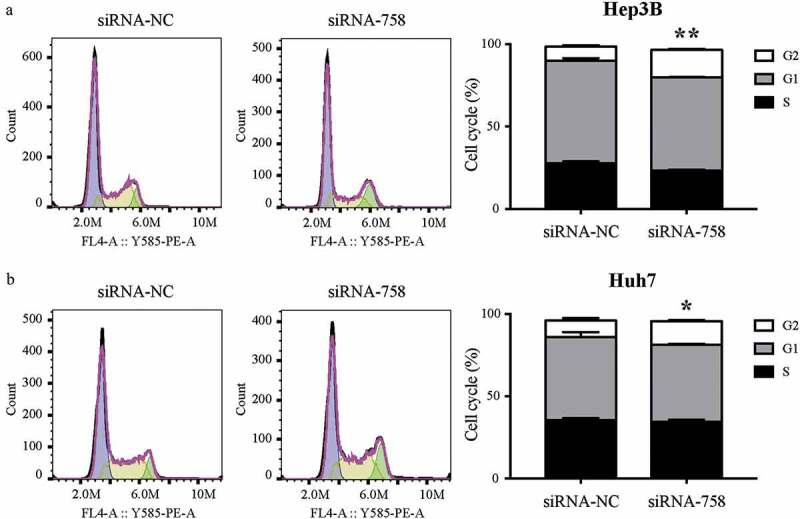
The distribution of the cell cycle in (a) Hep3B and (b) Huh7 cells was detected by flow cytometry. The number of cells arrested in G2/M phase was increased significantly in Hep3B (**P < 0.01) and Huh7 (*P < 0.05) cells.

The results of the cell cycle assay showed that the proportions of Hep3B and Huh7 cells in G2/M phase were 8.48% ± 1.07% and 9.95% ± 1.44% in the siRNA-NC group, while the rates were 16.77% ± 0.65% and 14.17% ± 0.81% in the siRNA-758 group. The statistical analysis indicated that the proportion of cells arrested in G2/M phase significantly increased in Hep3B (*P* < 0.01) and Huh7 (*P* < 0.05) cells (Figure 9). According to the results, we hypothesized that knocking down *CENPW* might prevent HCC cells from entering the mitotic phase.

### 3.9. Identification of CENPW associated DEGs

Normalized to the siRNA-NC group, 127 DEGs (82 upregulated and 45 downregulated) in the siRNA-758 group of Huh7 cells were identified (Figure S2). The upregulated DEGs included histone H2B (*HIST1H2BJ*) and cAMP-specific 3',5'-cyclic phosphodiesterase 7B (*PDE7B*), while downregulated DEGs included histone H4(*HIST4H4*), histone H2A(*HIST1H2AL*), histone H1.1(*HIST1H1A*), COMMD3-BMI1 readthrough (*COMMD3-BMI1*), 1-phosphatidylinositol 4,5-bisphosphate phosphodiesterase gamma-2 (*PLCG2*) and Complement C4-B (*C4B_2*).

### 3.10. GO biological process enrichment analysis

**Figure 10.**
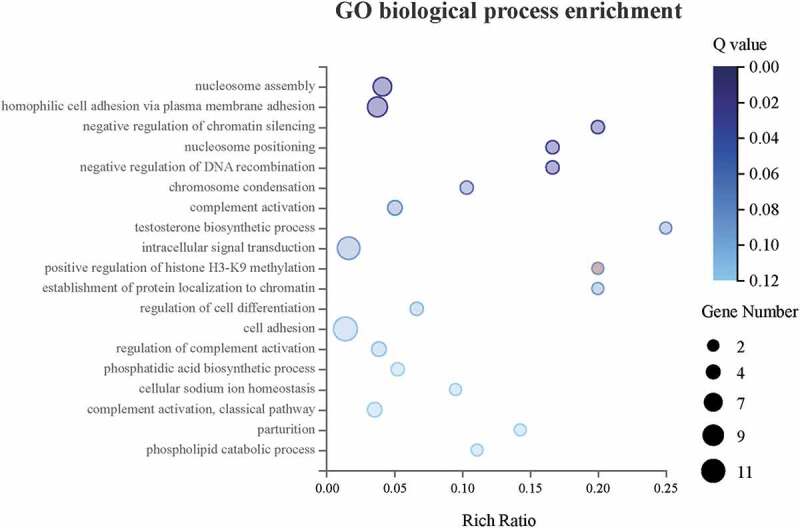
GO biological process enrichment analysis showed that the top 5 biological processes were significantly enriched (*Q*≤ 0.05) upon knockdown of *CENPW* in Huh7 cells.

To take the insight of the *CENPW* associated DEGs, GO biological process enrichment was performed (Figure 10). The DEGs were enriched in GO biological process significantly (*Q*≤ 0.05), including nucleosome assembly (GO: 0006334), homophilic cell adhesion via plasma membrane adhesion (GO: 0007156), negative regulation of chromatin silencing (GO: 0031936), nucleosome positioning (GO: 0016584), and negative regulation of DNA recombination (GO: 0045910).

### 3.11. KEGG pathway enrichment analysis

**Figure 11.**
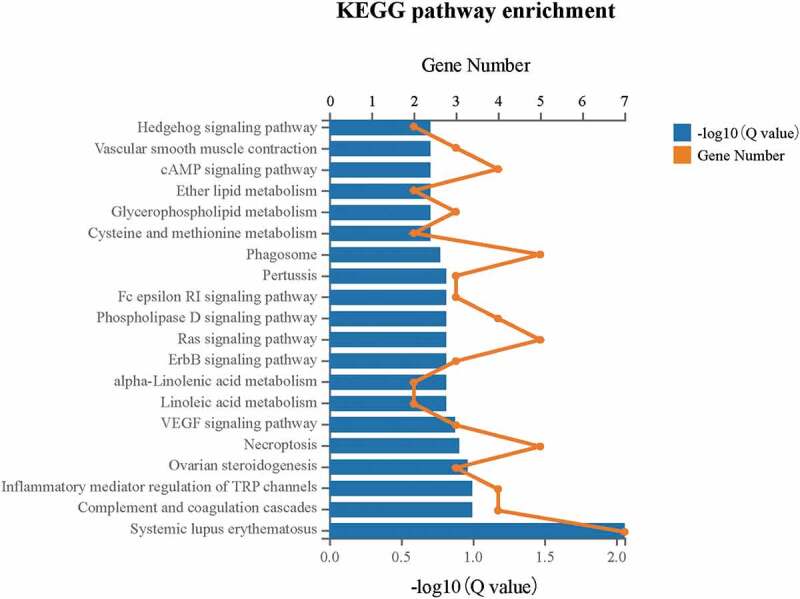
KEGG pathway enrichment analysis indicated that DEGs were significantly enriched in the systemic lupus erythematosus pathway (*Q*≤ 0.05) upon the downregulation of *CENPW* in Huh7 cells.

To identify the DEGs associated pathway, KEGG pathway enrichment was carried out (Figure 11). The DEGs were significantly enriched (Q ≤ 0.05) in the pathway of systemic lupus erythematosus (ID: 05322), which involved 7 DEGs including *HIST1H2AL, HIST1H2BJ, HIST4H4, C1 R, C4B_2, C7*, and *LOC110384692*. Moreover, the pathway of complement and coagulation cascades (ID: 04610) also had DEGs enrichment to a certain degree.

### 3.12. PPI network analysis

**Figure 12.**
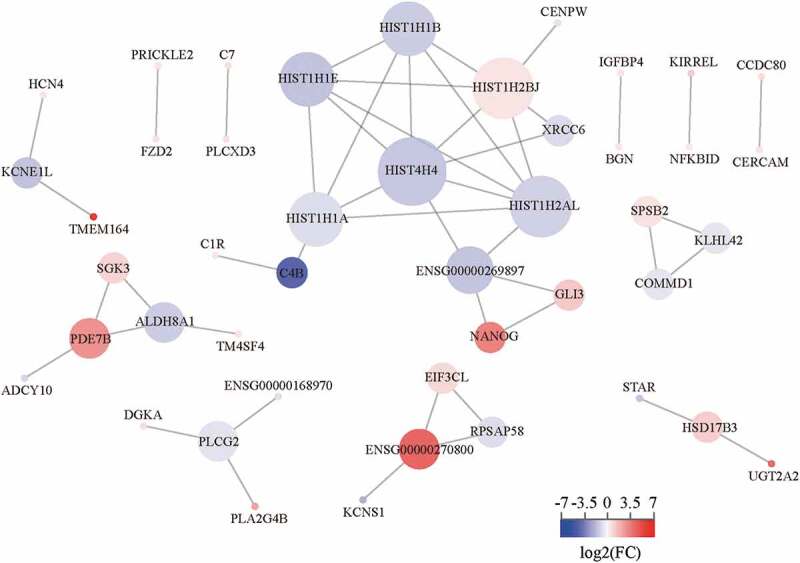
*CENPW* PPI network analysis in HCC. The size of different nodes indicated the degree centrality and the color of the nodes indicated the relative expression level of the DEGs.

We established a PPI network for 127 DEGs, containing 45 nodes and 48 edges (Figure 12). The DEGs were ranked respectively according to the three topological parameters, which were degree, closeness, and betweenness. The top 25 DEGs-coding proteins were listed (Table S1). We found that all the six histones ranked the top 25 in three topological parameters and occupied the hub nodes of the PPI network. Interestingly, we noticed the CENP-W directly interacted with the histone network through HIST1H2BJ, implying the CENP-W regulation of canonical nucleosomes or nucleosome-like structures. Moreover, C4B was also a hub node of the PPI network, indicating CENP-W might work viathe complement system.

### 3.13. qRT-PCR validation for RNA-seq data

**Figure 13.**
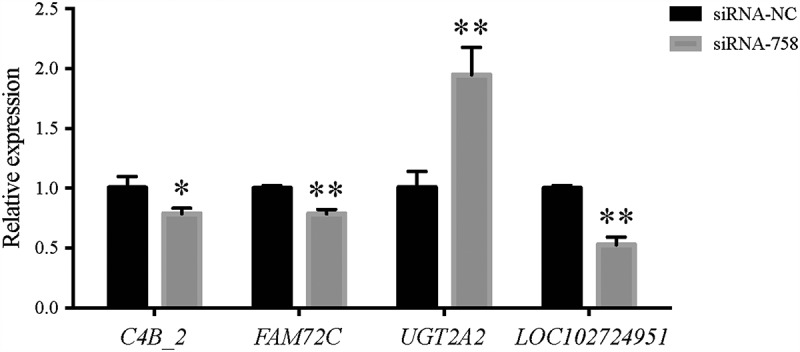
The qPCR detection showed that the expression of the *C4B_2, FAM72C, UGT2A2*, and *LOC102724951* genes in the siRNA-758 group was significantly different compared to the siRNA-NC group (*P < 0.05 and **P < 0.01), consistent with the RNA-seq data.

To validate the reliability of the RNA-seq data, we randomly selected four fromthe 127 DEGs to perform qRT-PCR detection. The random DEGs were *C4B_2, FAM72C, UGT2A2*, and *LOC102724951*. The primers were as follows: for the *C4B_2* gene: 5ʹ-GTGTGCATCTGGCGGAACG-3ʹ (forward), 5ʹ-TGGGGACCGAGTCAAAATACAG-3ʹ (reverse); for the *FAM72C* gene: 5ʹ-TCCTGTCTTCTTTCCTGCAACA-3ʹ (forward), 5ʹ-AGTAGGACGTTTACACCTGTGG-3ʹ (reverse); for the *UGT2A2* gene: 5ʹ-TGATGGCAAGACTTCAGAAAGG-3ʹ (forward), 5ʹ-TGTTACTGGGTCTGCTACCAA-3ʹ (reverse); and for the *LOC102724951* gene: 5ʹ-ACCACCGTAGTGGAAGAGAGA-3ʹ (forward), 5ʹ-GCTATGATGCCTATTTCCTCAGC-3ʹ (reverse). Consistent with the RNA-seq data, the expression of *C4B*_*2, FAM72C* and *LOC102724951* was confirmed to be downregulated, while that of *UGT2A2* was upregulated obviously (Figure 13).

Further, we investigated the differential expression of the reprogramming genes by qRT-PCR, such as *NANOG, OCT4, SOX2*, and *MYC*. The primers were as follows: for the *NANOG* gene: 5ʹ- TTTGTGGGCCTGAAGAAAACT-3ʹ (forward), 5ʹ-AGGGCTGTCCTGAATAAGCAG-3ʹ (reverse); for the *OCT4* gene: 5ʹ-CTTGAATCCCGAATGGAAAGGG-3ʹ (forward), 5ʹ-GTGTATATCCCAGGGTGATCCTC-3ʹ (reverse); for the *SOX2* gene: 5ʹ-GCCGAGTGGAAACTTTTGTCG-3ʹ (forward), 5ʹ-GGCAGCGTGTACTTATCCTTCT-3ʹ (reverse); for the *MYC* gene: 5ʹ-GGCTCCTGGCAAAAGGTCA−3ʹ (forward), 5ʹ-CTGCGTAGTTGTGCTGATGT−3ʹ (reverse). The expression of*NANOG, OCT4*, and *SOX2* were significantly increased (Figure S3), while that of *MYC* presented no statistical difference (data not shown).

## 4. Discussion

Previous studies showed that *CENPW* overexpression led to tumorigenesis and cell death, suggesting *CENPW* as a novel oncogene [20]. A recent study identified *CENPW* as one of the prognostic genes for HCC based on the gene co-expression network analysis, implying the prognostic value of *CENPW* for HCC [23]. However, the role of *CENPW* expression in the prognosis and development of liver cancer is still unknown. We confirmed the overexpression of *CENPW* in liver cancer using the Oncomine database. The results indicated that the *CENPW* expression level in HCC tissues was significantly higher than that in normal liver tissues (*P* < 0.01). In addition, the survival curves generated by the Kaplan-Meier plotter database showed that better prognosis of patients with liver cancer was correlated with lower *CENPW* expression. We suggest that *CENPW* is a potential predictive biomarker for liver cancer prognosis.

Centromere protein W (CENP-W), encoded by the *CENPW* gene, has three transcript variants. They are called isoforms a, b and c, with molecular weights of 11.8, 10.1 and 7.9 kDa, respectively. Isoform b is regarded as the canonical isoform, the most prevalent isoform in cells. In this article, we transiently knocked down *CENPW* expression in HCC cells by siRNA transfection. The siRNA-758 was designed to target the most conserved domain, which was shared by all three isoforms. In other words, all isoforms of CENP-W were knocked down after siRNA-758 transfection. In this way, the cellular behavior changes in the siRNA-758 group reflected all of the three CENP-W isoforms. Then, qRT-PCR and western blot were carried out to confirm the decrease in *CENPW* expression level. In Hep3B cells, 14.81% ± 3.26% of *CENPW* mRNA was left, while 27.14% ± 2.41% was left in Huh7 cells. The efficiency of siRNA-758 reached approximately 70% to 90%. A series of functional experiments in HCC cells implied that *CENPW* was an oncogene. Knocking down *CENPW* caused subsequent changes in HCC cells. The proliferation was obviously decreased from 24 to 72 h, and the cell apoptosis rate increased. The abilities of cell migration and invasion were also decreased significantly. In the cell cycle assay, the cells were arrested in G2/M phase, indicating that the cells had difficulty in mitosis after DNA synthesis. These results were reasonable, as *CENPW*is a centromeric component that plays a key role in cell division.

The results of RNA-seq and bioinformatic analysis showed that the *CENPW* downregulation mainly caused changes in nucleosome assembly, nucleosome positioning, and DNA and chromatin regulation, relating to histones H4, H2B, H2A, H1.1, H1.4, and H1.5 (Figure 12). Interestingly, only the expression of histone H3 among the nucleosome components has no significant difference. Moreover, we found that CENP-W interacted with nucleosome components directly through histone H2B (Figure 12), implying CENP-W and H2B might bridge the connection between centromere and nucleosome. Meanwhile, in the complement system, the expression of *C4B_2* was decreased, while that of *C7* and *C1R* was increased. It is worth further study of why *CENPW* is in close contact with the complement system. We also noticed the upregulation of the *NANOG* gene in the PPI network (Figure 12). By further confirmation using qRT-PCR, the transcription of the reprogramming factors (*NANOG, OCT4*, and *SOX2*) was enhanced by *CENPW* silencing in HCC cells (Figure S3), implying the possibility of reprogramming into cancer stem cells which characterized by inhibition of cell proliferation and metabolic changes [24,25]. Based on the analysis above, we hypothesized that CENP-W has biological functions other than its role as a centromeric component, and *CENPW* might be a potential predictive biomarker and a therapeutic target for liver cancer.

## 5. Conclusion

*CENPW* gene is a highly expressed oncogene in liver cancer, and is related to the prognosis of HCC patients, being a potential diagnostic biomarker in HCC. Based on our cellular experiments, the downregulation of *CENPW* expression might inhibit HCC development. Some important DEGs associated with *CENPW* knockdown were identified by RNA-seq, mainly correlating with nucleosomes and the complement system. These DEGs might be the *CENPW* downstream targets and potential therapeutic targets for HCC.

## Supplementary Material

Supplemental Material

## Data Availability

The data supporting the findings of this study are available within this article andthe supplementary materials.

## Highlights

- *CENPW* is highly expressed in liver cancer and correlated with the prognosis of HCC patients.
- *CENPW* might regulate the development of HCC by nucleosomes and the complement system.
- *CENPW* might be a potential predictive biomarker and the therapeutic target for HCC.
