# Supplementary material for: Histone-fold centromere protein W (CENP-W) is associated with the biological behavior of hepatocellular carcinoma cells
Source: Bioengineered. 2020 Jul 7;11(1):729–42. doi: 10.1080/21655979.2020.1787776 (PMC8291794; doi:10.1080/21655979.2020.1787776)
Supplement: Supplemental Material [file KBIE_A_1787776_SM8219.zip › Suppl table & fig caption.docx]

**Table S1**: The top 25 nodes in the topological parameters of the PPI network.

| Nodes | Degree | Nodes | Closeness | Nodes | Betweenness |
| --- | --- | --- | --- | --- | --- |
| HIST4H4 | 7 | HIST4H4 | 0.030178327 | ENSG00000269897 | 40 |
| HIST1H2BJ | 6 | HIST1H2AL | 0.030157642 | HIST1H1A | 40 |
| HIST1H2AL | 6 | HIST1H1A | 0.030095760 | HIST4H4 | 39 |
| HIST1H1A | 5 | HIST1H1E | 0.030095760 | HIST1H2BJ | 25 |
| HIST1H1E | 5 | HIST1H1B | 0.030095760 | HIST1H2AL | 24 |
| HIST1H1B | 5 | HIST1H2BJ | 0.030075189 | C4B | 22 |
| ENSG00000269897 | 4 | ENSG00000269897 | 0.030054646 | PLCG2 | 6 |
| PLCG2 | 3 | XRCC6 | 0.029993184 | ALDH8A1 | 6 |
| ALDH8A1 | 3 | C4B | 0.029911624 | PDE7B | 6 |
| PDE7B | 3 | GLI3 | 0.029850746 | ENSG00000270800 | 4 |
| ENSG00000270800 | 3 | NANOG | 0.029850746 | HIST1H1E | 3 |
| GLI3 | 2 | CENPW | 0.029850746 | HIST1H1B | 3 |
| SGK3 | 2 | C1R | 0.029689608 | KCNE1L | 2 |
| KCNE1L | 2 | ALDH8A1 | 0.024376731 | HSD17B3 | 2 |
| HSD17B3 | 2 | PDE7B | 0.024376731 | CCDC80 | 0 |
| EIF3CL | 2 | SGK3 | 0.024363233 | CERCAM | 0 |
| NANOG | 2 | TM4SF4 | 0.024336284 | KIRREL | 0 |
| KLHL42 | 2 | ADCY10 | 0.024336284 | NFKBID | 0 |
| COMMD1 | 2 | PLCG2 | 0.023809524 | IGFBP4 | 0 |
| SPSB2 | 2 | ENSG00000270800 | 0.023809524 | BGN | 0 |
| XRCC6 | 2 | EIF3CL | 0.023796646 | C7 | 0 |
| RPSAP58 | 2 | RPSAP58 | 0.023796646 | PLCXD3 | 0 |
| C4B | 2 | ENSG00000168970 | 0.023783784 | PRICKLE2 | 0 |
| CCDC80 | 1 | PLA2G4B | 0.023783784 | FZD2 | 0 |
| CERCAM | 1 | KCNS1 | 0.023783784 | GLI3 | 0 |

**Figure Legends**

**Figure S1**: *CENPW* transcription in subgroups of HCC patients based on gender, age, cancer stages, and tumor grades. Significant differences were observed in all of the subgroups when compared to the normal groups (**P*< 0.05, ***P*< 0.01 and ****P*< 0.001).

**Figure S2**: The volcano plot showed 127 significant DEGs (82 upregulated DEGs in red and 45 downregulated DEGs in blue) with |Log2(FC)| $\boldsymbol{\geq}$ 0.6 and FDR $\boldsymbol{\leq}$ 0.05 upon knockdown of *CENPW* in Huh7 cells.

**Figure S3**: The qRT-PCR analysis showed that the expression of the *NANOG*, *OCT4*, and *SOX2*genesin the siRNA-758 group was obviously upregulated compared to the siRNA-NC group (**P*< 0.05 and ***P*< 0.01) in Huh7 cells.
