# Supplementary figures and images for: Histone-fold centromere protein W (CENP-W) is associated with the biological behavior of hepatocellular carcinoma cells
Source: Bioengineered. 2020 Jul 7;11(1):729–42. doi: 10.1080/21655979.2020.1787776 (PMC8291794; doi:10.1080/21655979.2020.1787776)

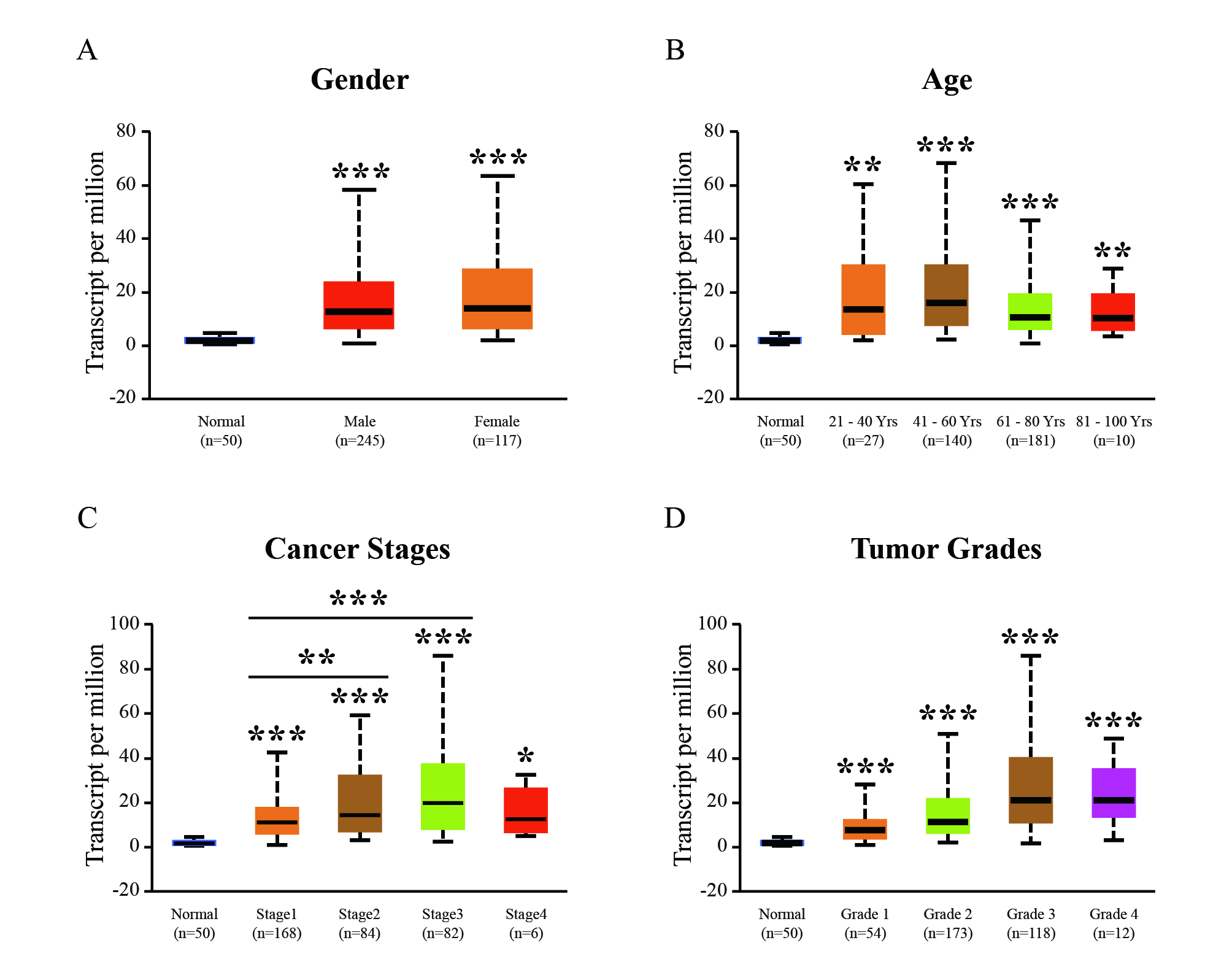

Supplement: Supplemental Material [file KBIE_A_1787776_SM8219.zip › FigS1.tif]

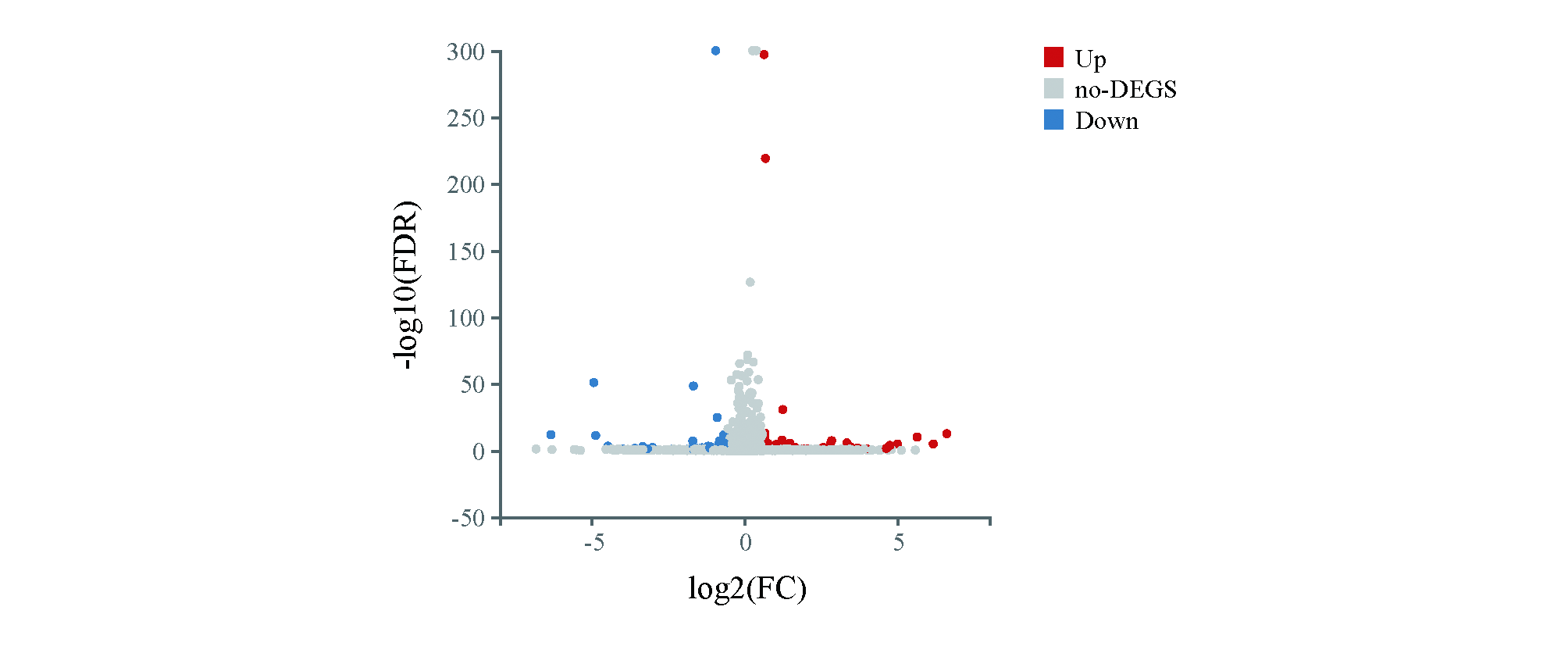

Supplement: Supplemental Material [file KBIE_A_1787776_SM8219.zip › FigS2.tif]

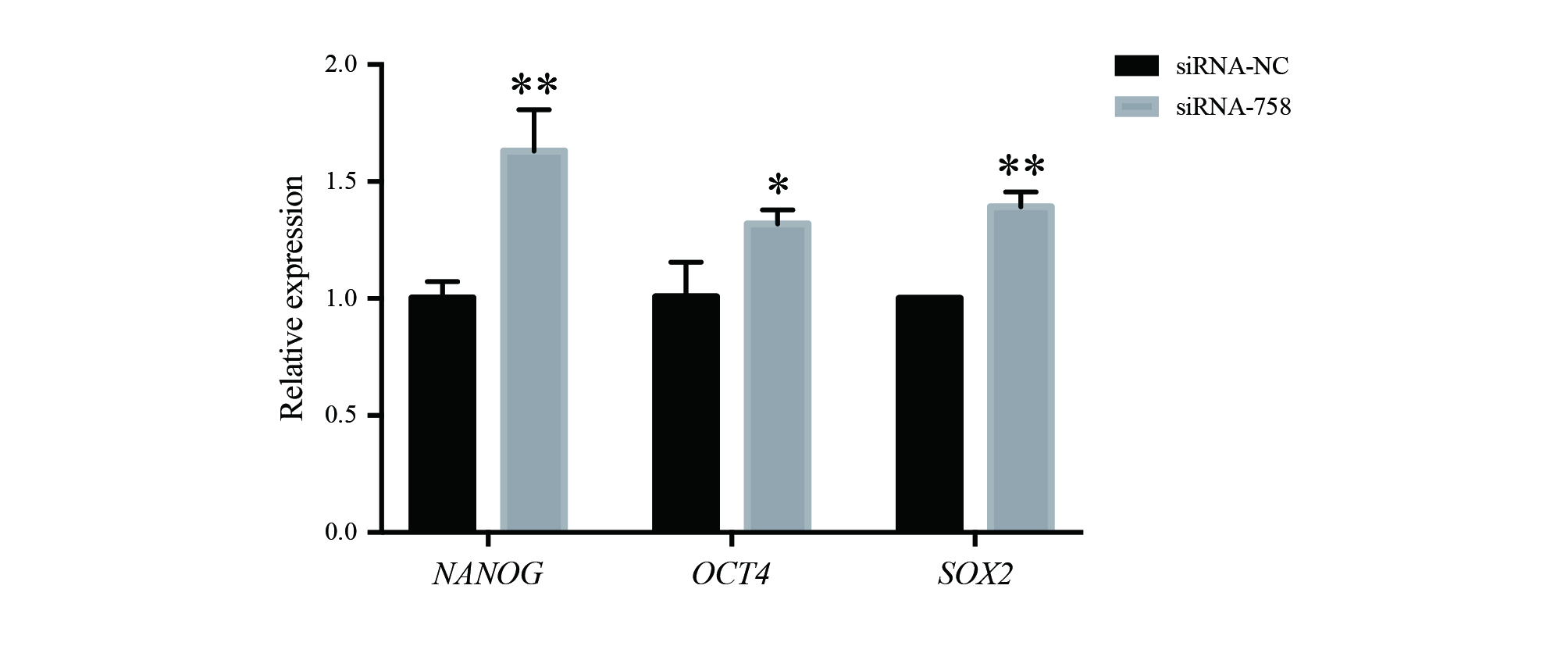

Supplement: Supplemental Material [file KBIE_A_1787776_SM8219.zip › FigS3.tif]
